# FastContext: A tool for identification of adapters and other sequence patterns in next generation sequencing (NGS) data

**DOI:** 10.18699/VJGB-22-97

**Published:** 2022-12

**Authors:** Е. Viesná, V. Fishman

**Affiliations:** Institute of Cytology and Genetics of the Siberian Branch of the Russian Academy of Sciences, Novosibirsk, Russia Novosibirsk State University, Novosibirsk, Russia; Institute of Cytology and Genetics of the Siberian Branch of the Russian Academy of Sciences, Novosibirsk, Russia Novosibirsk State University, Novosibirsk, Russia

**Keywords:** next generation sequencing, NGS, adapters, patterns search, read analysis, секвенирование нового поколения, NGS, адаптеры, поиск паттернов, анализ прочтений

## Abstract

The development of next generation sequencing (NGS) methods has created the need for detailed analysis and control of each protocol step. NGS library preparation protocols may include steps with incorporation of various service sequences, such as sequencing adapters, primers, sample-, cell-, and molecule-specific barcodes. Despite a fairly high level of current knowledge, during the protocol development process researches often have to deal with various kinds of unexpected experiment outcomes, which result either from lack of information, lack of knowledge, or defects in reagent manufacturing. Detection and analysis of service sequences, their distribution and linkage may provide important information for protocol optimization. Here we introduce FastContext, a tool designed to analyze NGS read structure, based on sequence features found in reads, and their relative position in the read. The algorithm is able to create human readable read structures with user-specified patterns, to calculate counts and percentage of every read structure. Despite the simplicity of the algorithm, FastContext may be useful in read structure analysis and, as a result, can help better understand molecular processes that take place at different stages of NGS library preparation. The project is open-source software, distributed under GNU GPL v3, entirely written in the programming language Python, and based on well-maintained packages and commonly used data formats. Thus, it is cross-platform, may be patched or upgraded by the user if necessary. The FastContext package is available at the Python Package Index (https://pypi.org/project/FastContext), the source code is available at GitHub (https://github.com/regnveig/FastContext).

## Abstract (ru)

Бурное развитие методов секвенирования нового поколения (next generation sequencing, NGS) породило потребность в детальном анализе и контроле качества на каждом этапе протокола приготовления геномных библиотек. Протоколы могут включать в себя этапы с внедрением различного рода служебных последовательностей, таких как адаптеры, праймеры, а также баркоды, специфичные для каждого образца, клетки или молекулы ДНК. Несмотря на достаточно высокий уровень современных знаний в молекулярной биологии, в процессе разработки протоколов NGS исследователи часто сталкиваются с неожиданными экспериментальными данными, которые могут быть результатом недостатка информации о молекулярных процессах, сопровождающих приготовление геномных библиотек, или, в отдельных случаях, дефектом производства реактивов. Обнаружение и анализ распределения служебных последовательностей в полученных молекулах ДНК могут быть важным источником информации, необходимой для оптимизации протокола приготовления геномных библиотек. В настоящей статье представлена утилита FastContext, с помощью которой возможен анализ структуры прочтений с точки зрения присутствия определенных последовательностей и их взаимного расположения в прочтении. Алгоритм принимает на вход необработанные данные секвенирования в формате FastQ, а затем генерирует удобные для интерпретации представления структуры прочтений на основе заданных пользователем паттернов, высчитывает количество подобных структур и их долю от общего числа прочтений. Несмотря на простоту алгоритма, FastContext может быть полезен при анализе структуры прочтений, он помогает лучше понять молекулярные процессы, происходящие на разных стадиях приготовления геномных библиотек и, как следствие, открывает возможности для усовершенствования протокола. FastContext – это проект с открытым исходным кодом, распространяемый под свободной лицензией GNU General Public License v3, полностью на- писанный на языке программирования Python и основанный на широко используемых программных пакетах и форматах данных. Таким образом, он может быть легко использован под любой операционной системой, ис- правлен и дополнен при необходимости. FastContext доступен в виде пакета в Python Package Index (https:// pypi.org/project/FastContext), исходный код хранится на GitHub (https://github.com/regnveig/FastContext).

## Introduction

Since the advent of next generation sequencing (NGS) methods 20 years ago, those methods have been actively evolving and are currently applied to various areas of biology. Due to the increasing capacity of sequencers, it is now possible to obtain billions of short molecule sequences in a single NGS run. In order to utilize such a high throughoutput of modern sequencers, there is a practice of sample pooling. This method requires incorporation of sample-specific service sequences (barcodes), which allow to distinguish individual samples in raw sequencing data.

Other types of service sequences could be incorporated into the target molecules, such as sequencing adapters and primers, biotin-labeled oligonucleotides for target molecules enrichment (Gridina et al., 2021), molecule- and cell-specific barcodes, which are designed to identify a molecule (Smirnov et al., 2020) and/or a cell of origin (Aldridge, Teichmann, 2020).

There are many strategies in molecular genetics that are used for service sequences incorporation: direct ligation of DNA or RNA molecules, template-switching activity of reverse transcriptases, and incorporation of synthetic DNA transposons. During the whole process of new NGS methods development it is crutial to control each protocol step. In light of that, detection and analysis of service sequences distribution may provide important information for protocol optimization.

Here we introduce the FastContext tool, which is designed to analyze and compute statistics on NGS read structures. FastContext allows to search for user-specified sequences in NGS reads, gather data on their linkage, frequency of occurence, and present statistics in a user-friendly manner.

## Materials and methods

The script is completely written in the programming language Python (version 3.8). It is packaged as a part of the Python Package Index (https://pypi.org/project/FastContext) and can be installed via pip. Therefore, it works out of the box on every operating system.

We used the following Python libraries: 1. bioPython, version 1.79 (Cock et al., 2009): FastQ files parsing and sequences manipulation; 2. python-Levenshtein (Available at: https://github.com/ztane/python-Levenshtein), version 0.12.2: calculating sequences Levenshtein distance; 3. pandas, version 1.2.5 (The Pandas Development Team, 2020): tables creation; 4. tqdm, version 4.61.2 (Costa-Luis et al., 2022): visualization.

All libraries listed above, except python-Levenshtein, are widely used and well maintained.

FastContext supports multi-processing

## Results

**Fig. 1.**
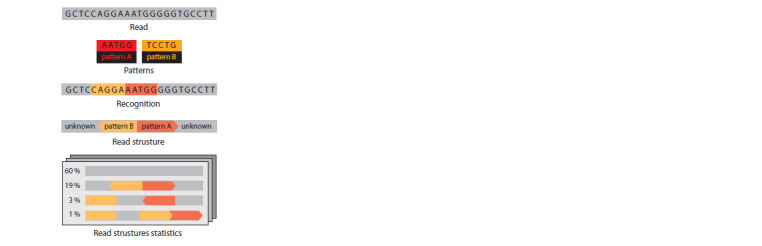
FastContext algorithm scheme Two different example patterns colored as red and yellow

We developed an algorithm which parses raw sequencing dataset, searches each read or read pair for user specified patterns, and then generates a human-readable representation of the search results, which we call “read structure”. Algorithm scheme is represented in the Fig.1.

Input

Input files are provided in FastQ format (Full specification of FastQ format is available at http://maq.sourceforge.net/ fastq.shtml). The user can provide one (in the single-end mode) or two (in the paired-end mode) FastQ files. Files may be uncompressed or compressed with gzip or bz2 algorithms.

Output

**Fig. 2.**
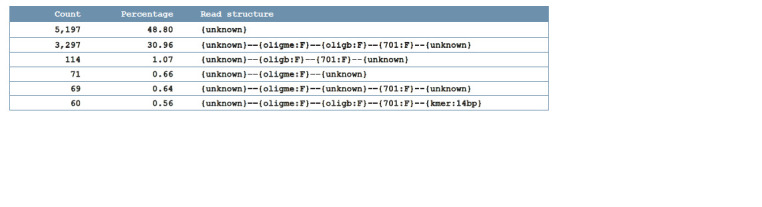
Example of statistics table. Every fragment of read structure, except palindromic or unrecognized sequences, has a strand suffix. Short unrecognized sequences (K-mers) have a length suffix.

Output results are provided as an HTML page (further: “summary file”), containing run options and tables with read structures, their counts, and percentages (Fig. 2). The sequence strand (forward F, or reverse R) is displayed after a colon (e. g., {oligb:F}).

The user can manually set minimal rate value (rate floor) to be displayed. Also, the user can save the read structure for each read or read pair, with the read name, the sequence, and Phred qualities, as a gzip-compressed JavaScript Object Notation (JSON) object (further: “detailed statistics file”)Full specification of JSON format could be found at JSON official website: https://www.json.org..

Patterns

Pattern names and sequences are provided as a plain JSON object, e. g.: { foo": "CTGTCTCTTATACAC", "bar": "CCGAAAACACG", "baz": "TCGTCGGG"}. It should be noted that pattern sequences are searched in the order they are provided by the user, forward strand (the sequence itself) first, reverse strand (a reverse complement of the sequence) after. Therefore, the patterns order matters in search and should be carefully considered before running the program. FastContext expects patterns to be sorted from long to short, which is the best option for overlapping or nested sequences, and otherwise gives you a warning.

K-mers

FastContext performs the search based on full match, and a pattern sequence with one single sequencing error will be skipped as an unrecognized sequence (alias {unknown}). This is especially important for long patterns, which are underrepresented due to higher cumulative frequency of sequencing errors. In addition, oligonucleotide synthesis errors and some enzymatic steps of NGS library preparation, such as A-tailing, may produce molecules one base pair shorter or longer than expected. In order to simplify identification of such extended or truncated sequences, we have implemented the ability to mark short unrecognized sequences (K-mers) of certain length (e. g., {kmer:14bp}). If a K-mer identified in the read is one base longer or shorter than a pattern sequence, we can suppose this K-mer is the pattern sequence, and test the hypothesis in a more detailed analysis of reads.

Levenshtein distances analysis

Additional features implemeted to account for sequencing errors include analysis of Levenshtein distances between different pattern sequences (pattern analysis), and between pattern sequences and read sequence. Pattern analysis is shown in the summary file, data on every single read can be found in the detailed statistics file.

Analysis of distances between pattern sequences can prevent pattern match or nesting, when sequences are confused with each other because of a few sequencing errors. Also, FastContext warns the user about palindromes and sequences that can become palindromic because of sequencing errors. This kind of sequence may affect statistics of forward-reverse orientation.

Analysis of distances between pattern and read sequences can show similarity of an unrecognized sequence and a pattern sequence, so the user could suggest the real read structure even if FastContext fails to do that. All these data may be found in a detailed statistics file, with Levenshtein read analysis enabled (disabled by default).

System requirements and performance

By design, FastContext stores FASTQ reads in random access memory (RAM), therefore, the only system limitation is the RAM size. Tests we have performed show that 8 Gb RAM is enough for processing 10,000 reads, which is a high enough sample size for practical application of the tool.

There are two stages that determine the time taken for completing a task. Reading data from a physical storage (HDD, SSD, etc.) depends on the storage characteristics. Read analysis is parallelized and depends on the core number. We estimated FastContext performance characteristics on the laboratory computing server with 16 cores and 50 Gb RAM. The dependence of processing speed on process count matches the expected values. 10,000 of paired-end reads are processing for 2 seconds with 4 cores used, saving JSON increases that time to 6 seconds. With Levenshtein statistics, the same data are processing for 11 seconds, and 80 seconds are required to save JSON.

Code access

FastContext source code is available at GitHub (https://github. com/regnveig/FastContext) and is distributed under GNU General Public License v3.

## Discussion

Despite the simplicity of the algorithm, FastContext may be useful in read structure analysis. It has an appealing combination of cutadapt (Martin, 2011) and FastQC (Andrews, 2010) features

Recently, A. Bravo et al. (2021) presented a tool named 2FAST2Q, which has features similar to FastContext, including extracting and counting feature occurrences in FastQ files. Unlike FastContext, 2FAST2Q can search for frequent unknown sequences (so called extract and count mode), can handle sequence mismatches, takes into account base Phred qualities, and therefore provides more accurate statistics on feature counts. The qualitative difference of FastContext is that the tool can collect statistics on relative position of features in the read and features linkage.

There remains the problem of sequencing errors. The possibility of errors is directly dependent on sequence length. FastContext performs the search based on full match, there fore, under equal conditions, pattern sequences of greater length have a lower chance to be found, which may impact resulting statistics.

Similarity based on Levenshtein distance is a crude approximation to probability of presence of a particular sequence. It fails to take account of in vitro processes during library preparation and sequencing. This problem may be solved in future versions. As for now, the user can find Phred quality scores for each read in a detailed statistics file, and estimate analysis quality manually.

Another possible feature that can be discussed is wildcards (symbols which denote more than one canonical nucleobase). This feature may be implemented in future versions.

## Conclusion

From all of the above, we can conclude that FastContext is effective as a tool for NGS data analysis, and could be a very useful source of information in the development of new molecular biology methods.

## Conflict of interest

The authors declare no conflict of interest.
